# Endoscope-assisted resection of a brainstem cavernoma

**DOI:** 10.3171/2019.7.FocusVid.19158

**Published:** 2019-07-01

**Authors:** Florian Roser, Luigi Rigante, Mohamed Samy Elhammady

**Affiliations:** Department of Neurosurgery, Neurological Institute, Cleveland Clinic Abu Dhabi, United Arab Emirates

**Keywords:** cavernous malformation, brainstem, endoscope, video

## Abstract

Procedures on cavernous malformations of the brainstem are challenging due to their eloquent location. This accounts especially for recurrent cavernomas as surgical scars, adhesions, and functional shift might have occurred since primary surgery. We report on a 38-year-old female patient with a large recurrent brainstem cavernoma, who underwent previous successful surgery and experienced recurrent bleeding about 2 years later. She harbored a large associated developmental venous anomaly (DVA) traversing the cavernoma through the midline of the brainstem. In order to visualize complete resection and preservation of the DVA at the same time, endoscopic-assisted resection within the brainstem after decompression in the semisitting position was performed.

The video can be found here: https://youtu.be/K1p-Sx7jUpA.

## Transcript

This is a 43-year-old female patient who underwent microsurgical resection of a brainstem cavernoma about 18 months earlier with no residual deficit. She appears again with new swallowing deficit, sixth and seventh nerve palsy on the right side, as well as heminumbness and acute hemiparesis of the left side. There is a large bleeding cavity again noted in the brainstem as well as an associated DVA traversing the midline of the brainstem.

0:51 Surgery was planned in the semisitting position under intraoperative nerve monitoring for SSEPs, MEPs, BAEP, as well as EMG and MEP sixth nerve, seventh nerve, and lower cranial nerves.
1:11 Surgery was planned about 2 weeks after the acute onset of new symptoms to allow liquefication of the hematoma and easier mobilization of the hematoma within the brainstem.
1:25 The semisitting position has several advantages as it allows drainage and irrigation at the same time with less manipulation of the bleeding cavity.
1:44 The former right-sided retrosigmoid craniotomy was reopened, removal of the bone flap visualization of transverse and sigmoid sinus. Tacking sutures towards lateral on the opened dura and detachment of the cerebellum of the dura. Retraction of the cerebellum against gravity and adhesiolysis of the scar tissue at the cerebellopontine angle under high magnification of the microscope.
2:40 In the left hand you see the suction-irrigation tool which allows dissection, irrigation, and flushing at the same time. Visualization of the seventh/eighth nerve bundle arachnolysis there. The more arachnolysis the better, to allow retraction and visualization of the cerebellopontine angle, especially the entry zone into the BS.
3:00 Here you see the lower cranial nerves and the choroid plexus at the lateral recess. There is adhesion from the former surgery of the vertebral artery at the brainstem, which will be sharply dissected to allow mobilization as well.
3:50 With neuronavigation and monopolar mapping identification of the optimal entry point into the brainstem in an avascular zone of former entry. Pinpoint coagulation to allow opening of the very thin pial membrane to the bleeding cavity. Now the suction-irrigation device is of much advantage in the sitting position. Opening of the hematoma cavity and flushing out the liquefied hematoma with as less manipulation as possible. Real-time electrophysiological monitoring of the cranial nuclei and the long tracks allow surveillance during manipulation and resection of the brainstem cavernoma. After complete removal of the liquefied hematoma parts, the more firm cavernoma parts can be gently mobilized and pulled out.
5:20 This allows at the end of the resection visualization of the brainstem traversing associated DVA in the midline of the brainstem. Here no further resection should be forced as this vein should be preserved by all means.
7:00 After microsurgical resection of the cavernoma the DVA again is inspected and a 30° angled endoscope is inserted into the cerebellopontine angle and into the resection cavity of the brainstem under continuous irrigation and flushing to allow an open brainstem cavity. Here the DVA again is noted along its course through the brainstem as well as remaining cavernoma parts, which again can then be resected either under endoscopic assistance or by microsurgical techniques. The retrosigmoid craniotomy is then closed in a standard fashion with refixation of the bone flap with miniplates; the cavernoma was completely resected; the DVA preserved. The patient made an uneventful recovery with complete resolution of the swallowing deficit and some remaining hypoesthesia.
